# A blockchain-enabled trust-aware authentication framework for secure communication in VANETs

**DOI:** 10.1038/s41598-026-51446-6

**Published:** 2026-06-20

**Authors:** S. Sajini, V. Jananee, E. A. Mary Anita

**Affiliations:** 1 Department of Computer Science and Engineering with Specialization in Artificial Intelligence and Machine Learning, SRM Institute of Science and Technology, Ramapuram Campus, Chennai, India; 2 Department of Computer Science and Engineering, School of Engineering and Technology, Christ University, Bengaluru, India

**Keywords:** Vehicular Ad Hoc Networks, Blockchain-based authentication, Trust-aware security, Lightweight cryptography, Deep learning-based trust evaluation, Cluster-based secure communication, Intelligent transportation systems, Engineering, Mathematics and computing

## Abstract

Vehicular Ad Hoc Networks (VANETs) are essential for intelligent transportation systems; however, their dynamic topology and open wireless communication environment expose them to impersonation, Sybil, replay, and message modification attacks. Existing authentication schemes mainly rely on centralized authorities and conventional cryptographic mechanisms, which lack dynamic trust evaluation and fail to ensure secure authentication during Roadside Unit (RSU) handover and cluster mobility. To address these challenges, this paper proposes a blockchain-enabled trust-aware authentication framework for secure VANET communication. The framework integrates a Joint Probability and Error Unit-based Deep Learning Neural Network (JPEU-DLNN) for dynamic trust assessment and Log-based Edwards Curve Cryptography (LECC) for lightweight key generation. In addition, Gini Indexed Farthest First Clustering (GIFFC) ensures stable cluster formation, while the Directional Gannet Optimization Algorithm (DGOA) supports optimal routing. Blockchain technology provides decentralized and immutable credential storage, eliminating single-point failure and certificate forgery. Simulation results show that the proposed framework achieves 95.62% authentication accuracy and 96.94% packet delivery ratio, with reduced end-to-end delay and improved network lifetime compared to existing blockchain-based authentication schemes. Security evaluation confirms strong resistance against active and passive attacks. These results demonstrate the practical applicability of the framework for secure and reliable VANET deployments.

## Introduction

In recent years, **Vehicular Ad Hoc Networks (VANETs)** have emerged as a focal point within intelligent transportation systems (ITSs) because of their significant potential to enhance road safety and driving efficiency^1^. VANET communication typically occurs in three modes: **Vehicle‑to‑Vehicle (V2V)**,** Vehicle‑to‑Infrastructure (V2I)**,** and Vehicle‑to‑Roadside Unit (V2R)**, each enabling the exchange of safety and general information across the network^2^. Through On‑Board Units (OBUs), vehicles transmit safety messages that help mitigate accidents and congestion by ensuring critical traffic information is shared among participants^3^.

Despite these benefits, VANETs also present notable **security and privacy challenges**. The exchange of sensitive data exposes users to risks of identity theft and misuse when exploited by malicious actors^4^. Among the various threats, the **Sybil attack** is particularly severe, as adversaries generate multiple false identities to disrupt network functionality^5^. While cryptographic techniques have been widely adopted to secure VANETs, reliance solely on encryption and hashing has proven insufficient, leaving vulnerabilities unresolved^6^. To address these limitations, **blockchain technology** has recently been integrated into VANET frameworks, offering decentralized trust, secure data sharing, and robust authentication mechanisms^7^. By combining blockchain with VANET protocols, researchers aim to strengthen privacy and reliability in vehicular communications^8^.

Blockchain stores information of vehicular nodes as blocks that are chained together and it is not possible to update or delete any information from the blocks. One of the defining characteristics of **blockchain technology** is its decentralized nature, which eliminates the need for third‑party intermediaries in transaction verification. Every participant maintains a complete copy of the ledger, and once a new block is appended, all nodes update their records accordingly^9^. Although several blockchain‑based authentication frameworks have been introduced, many still fall short of meeting the stringent security and privacy demands of VANETs, particularly in areas such as entity verification and message authentication. These approaches often suffer from limitations related to computational efficiency and communication overhead^10^. Consequently, there is a pressing need for a **robust trust management system** and secure communication framework capable of mitigating insider threats. To address this gap, the present work proposes a **blockchain‑enabled authorization scheme** that integrates JPEU‑DLNN‑based trust evaluation with LECC‑based security, specifically designed for V2V communication in VANETs.

### Efficiency problem in VANET

Due to high vehicle mobility and dynamic topology in Vehicular Adhoc Networks (VANETs), existing authentication mechanisms suffer from increased computational overhead, communication delay, and centralized trust dependence. These issues lead to authentication latency and reduced scalability in real-time vehicular environments. Therefore, designing an efficient lightweight authentication mechanism with reduced computation cost and fast session key agreement is essential for secure vehicle-to-infrastructure communication.

### Problem statement

Despite advancements in authentication mechanisms for Vehicular Ad Hoc Networks (VANETs), existing solutions fail to provide a unified framework that ensures decentralized authentication, dynamic trust evaluation, lightweight cryptographic efficiency, and secure mobility support simultaneously. Centralized approaches suffer from single-point failure and scalability limitations, while existing blockchain-based schemes primarily focus on credential storage without addressing real-time trust assessment and authentication continuity during cluster transitions and Roadside Unit (RSU) handovers.

Moreover, high vehicle mobility and open wireless communication channels increase vulnerability to Sybil, impersonation, replay, and man-in-the-middle attacks. The absence of an integrated mechanism that combines decentralized credential management, intelligent trust evaluation, and mobility-aware authentication leads to reduced reliability and increased security risks in VANET environments.

Therefore, the fundamental problem addressed in this study is the lack of a comprehensive, decentralized, and trust-aware authentication framework capable of ensuring secure, lightweight, and mobility-resilient communication in VANET systems.

The overall structure of this paper is organized as: Sect. 2 presents the literature survey, Sect. 3 details the proposed techniques, Sect. 4 discusses the experimental results, and Sect. 5 concludes with recommendations for future improvements.

### Objectives of the study

The primary objectives of this study are as follows:

1. To design a decentralized authentication framework for Vehicular Ad Hoc Networks (VANETs) that eliminates single-point failure and enhances credential security.
2. To develop a trust-aware mechanism capable of dynamically identifying malicious and legitimate vehicles in highly mobile environments.
3. To reduce computational and communication overhead through the use of lightweight cryptographic techniques suitable for resource-constrained vehicular nodes.
4. To improve authentication continuity during cluster transitions and Roadside Unit (RSU) handover.
5. To evaluate the performance of the proposed framework in terms of authentication accuracy, packet delivery ratio, delay, and resistance to common security attacks.

## Literature survey

Maria et al. (2022)^11^ proposed the **Blockchain‑based Anonymous Authentication and Integrity Preservation (BAIV)** scheme for VANETs. In this approach, blockchain was integrated into VANET to enable vehicle authentication without relying on a centralized trusted authority. Their performance evaluation demonstrated the effectiveness of BAIV in ensuring secure communication. However, the scheme required each node to authenticate messages individually, which introduced significant delays in message transmission.

In another study, Maria et al. (2021)^12^ introduced the **Blockchain‑Based Anonymous Authentication Scheme (BBAAS)** to enhance secure communication in VANETs. In this model, **Roadside Units (RSUs)** were responsible for authenticating vehicles, and subsequent communications were secured using shared session keys. The analysis confirmed that BBAAS was cost‑efficient. Nevertheless, the scheme did not adequately address security concerns when vehicles moved between different coverage regions, leaving a gap in continuous authentication.

Alharthi et al. (2021)^13^ proposed a **Biometrics Blockchain (BBC)** framework aimed at enhancing privacy in VANETs. In this model, biometric data was used to record the genuine identity of message senders, thereby ensuring privacy preservation. Their evaluation showed improved performance compared to existing schemes. However, the framework did not address malicious message handling, which could undermine its effectiveness.

D. Zhang et al. (2022)^14^ introduced a **Blockchain‑based Multi‑Access Edge Computing for Future VANETs (BMEC‑FV)**. This approach employed multiple MEC servers to calculate trust among vehicles through computation offloading, while a deep compressed neural network was applied to solve optimization problems. Despite its strengths, the absence of attack analysis raised concerns about its overall security.

Sajini & A (2022)^15^ developed a framework to strengthen secure data communication in VANETs. Their method utilized an **ASCII‑ECC (American Standard Code for Information Interchange‑centered Elliptic Curve Cryptography)** scheme. Performance results indicated superior efficiency compared to other models. Nonetheless, when a cluster head (CH) migrated between clusters, delays in selecting a new CH increased communication time.

Kudva et al. (2021)^16^ designed a **scalable blockchain‑based trust management protocol** for VANET routing. The system incorporated a two‑level insider attack detection mechanism, combining individual trust computation with consortium blockchain aggregation. Experiments confirmed scalability and efficiency for large networks. Yet, the lack of V2V communication limited data sharing capabilities.

H. Li et al. (2020)^17^ presented a **Fine‑grained Access Control scheme for VANET Data (FADB)**, integrating blockchain with ciphertext‑based attribute encryption. Simulations demonstrated effective data security with low overhead. However, the scheme remained vulnerable to insider threats due to malicious message injection.

Inedjaren et al. (2021)^18^ implemented a **blockchain‑based distributed trust management system** using the Optimized Link State Routing (OLSR) protocol. Results showed reduced detection time and overhead. Still, as the number of nodes increased, control message overhead also grew, affecting scalability.

Jenefa & Anita (2021)^19^ proposed an **identity‑based message authentication scheme** using proxy vehicles. Their ECC‑based approach (without pairings) allowed proxy vehicles to verify multiple messages, achieving high throughput (e.g., RSUs verifying over 226,000 messages per second). However, the scheme did not address fake message attacks, which could degrade performance.

Lin et al. (2021)^20^ introduced the **Blockchain‑based Conditional Privacy‑Preserving Authentication (BCPPA)** protocol. By combining blockchain with ECDSA‑based certificate management, the model improved security and utility. Nevertheless, communication costs were higher compared to other schemes.

B. Li et al. (2020)^21^ suggested a **blockchain‑based trust management model** for location privacy in VANETs. Vehicles used certificates to request location services, while blockchain secured data exchanges. Experiments showed resilience against trust model attacks. Yet, the system faced limitations due to potential location privacy leakage.

Al‑shareeda & Anbar (2020)^22^ examined a **privacy‑preserving communication scheme** for VANETs that combined ECC with identity‑based encryption. Security analysis revealed vulnerabilities, particularly due to weaknesses in elliptic curve base point selection, leaving the scheme exposed to external attacks.

Nandy et al. (2021)^23^ proposed a **lightweight ECC‑based authentication scheme** for VANETs. Performance analysis highlighted reduced energy costs and improved efficiency. However, the random generation of authentication keys weakened the model’s security.

Alfadhli et al. (2020)^24^ implemented the **Safe Driving and Privacy‑Preserving Authentication (SD2PA)** scheme, which relied on hashing and anonymity features to protect vehicle privacy. Communication overhead analysis showed improvements over related schemes. Still, reliance on TA authentication introduced re‑authentication delays, potentially reducing network lifetime.

Su et al. (2022)^25^ developed the **Trusted Blockchain‑based SignCryption and Data Management (TB‑SCDM)** protocol. The scheme included multiple phases such as initialization, signcryption, authentication, and transaction management. Results indicated reduced storage requirements. However, authentication was not maintained when nodes moved between regions, leaving the system vulnerable to attacks.

Recent advancements in blockchain-enabled security frameworks for VANETs have demonstrated significant improvements in authentication, privacy preservation, and trust management. For instance, recent studies have explored lightweight authentication mechanisms integrated with blockchain to ensure secure vehicle-to-vehicle (V2V) communication while reducing computational overhead. Similarly, trust-aware frameworks leveraging distributed ledger technologies have been proposed to mitigate malicious node behavior and ensure data integrity in highly dynamic vehicular environments.

Furthermore, contemporary works have focused on enhancing scalability and latency performance using optimized consensus mechanisms and edge-assisted blockchain architectures. Some approaches also incorporate machine learning techniques to improve anomaly detection and adaptive trust evaluation in VANETs. Despite these advancements, many existing solutions either rely heavily on centralized components, incur high communication overhead, or lack comprehensive trust evaluation mechanisms under real-time vehicular conditions.

In contrast, the proposed framework introduces a blockchain-enabled trust-aware authentication model that integrates decentralized trust management with efficient authentication protocols, ensuring enhanced security, reduced latency, and improved scalability. The proposed system uniquely combines trust evaluation with blockchain immutability, thereby addressing limitations observed in existing approaches.

**Table 1:** Comparative analysis of existing blockchain-based VANET authentication schemes.

References	Approach	Blockchain usage	Trust mechanism	Security strength	Latency	Key limitation
^26^ Salami et al. (2026)	Blockchain-based secure data offloading using Fog-Edge federation	Yes	No	High	High	High computational overhead in UAV-assisted environments
^27^ Salami et al. (2024)	Secure task offloading in Fog-Cloud-based IoV	Partial	No	Moderate–High	Medium	Absence of integrated trust evaluation
^28^ Zhang et al. (2024)	Blockchain-based VANET framework	Yes	Yes	Limited	Medium	Scalability issues
^29^ Salami et al. (2021)	Cost-effective secure key exchange scheme (CE-SKE)	No	No	High	Low	Lack of decentralization due to absence of blockchain
^30^ Sharma et al. (2023)	Trust-aware VANET routing	Yes	Yes	Limited	High	High Latency
^31^ Salami et al. (2024, IEEE)	Lightweight secure mutual authentication and key agreement (LSMAK-IOV)	No	Partial	High	Low	Limited scalability in highly dynamic VANET environments
Proposed Work	Blockchain-enabled trust-aware authentication with clustering	Yes	Yes (Integrated)	High	Low	—

As observed from Tables 1, 2, existing approaches primarily focus on secure data offloading, key exchange, and authentication mechanisms. However, most of these methods lack integrated trust evaluation and decentralized security using blockchain. Additionally, some approaches introduce higher computational overhead or fail to scale efficiently in dynamic vehicular environments. In contrast, the proposed framework combines blockchain technology with trust-aware authentication and efficient clustering, enabling improved security, reduced latency, and enhanced scalability in VANET scenarios^32–35^.

### Research gap analysis

A comprehensive review of recent authentication schemes in VANET environments reveals several limitations. Traditional cryptographic approaches, such as RSA and Elliptic Curve Cryptography (ECC)-based schemes, provide secure key exchange mechanisms but rely heavily on centralized authorities, leading to scalability issues and single-point failure risks.

Recent blockchain-based authentication frameworks address decentralization; however, most existing solutions focus primarily on credential storage and integrity verification. These approaches often overlook dynamic trust evaluation mechanisms necessary for identifying malicious behavior in highly mobile VANET scenarios.

Furthermore, many existing schemes do not adequately address authentication continuity during cluster transitions and Roadside Unit (RSU) handover. High computational overhead and lack of lightweight cryptographic optimization also remain significant concerns for resource-constrained vehicular nodes.

Therefore, a clear research gap exists in developing an integrated framework that simultaneously ensures decentralized authentication, dynamic trust assessment, lightweight cryptographic efficiency, and mobility-aware clustering. The proposed blockchain-enabled trust-aware authentication framework is designed to address these unresolved challenges.’

**Table 2:** Research gap identification.

Refs.	Decentralized	Trust evaluation	Lightweight crypto	Mobility support	Gap identified
[R1]	✔	✖	✖	✖	No trust model; centralized dependency
[R2]	✔	✖	✔	✖	No mobility-aware authentication
[R3]	✔	✔	✖	✖	High computational overhead
[R4]	✔	✖	✔	✖	No RSU handover continuity
Proposed	✔	✔	✔	✔	Integrated decentralized, trust-aware, lightweight, mobility-resilient framework

Existing works address decentralization or trust mechanisms individually; however, none provide an integrated framework combining blockchain-based authentication, dynamic trust evaluation, lightweight cryptography, and mobility-aware support. This identified gap motivates the proposed framework.

## Proposed blockchain authorization-based communication methodologies in vanet

**Fig. 1:**
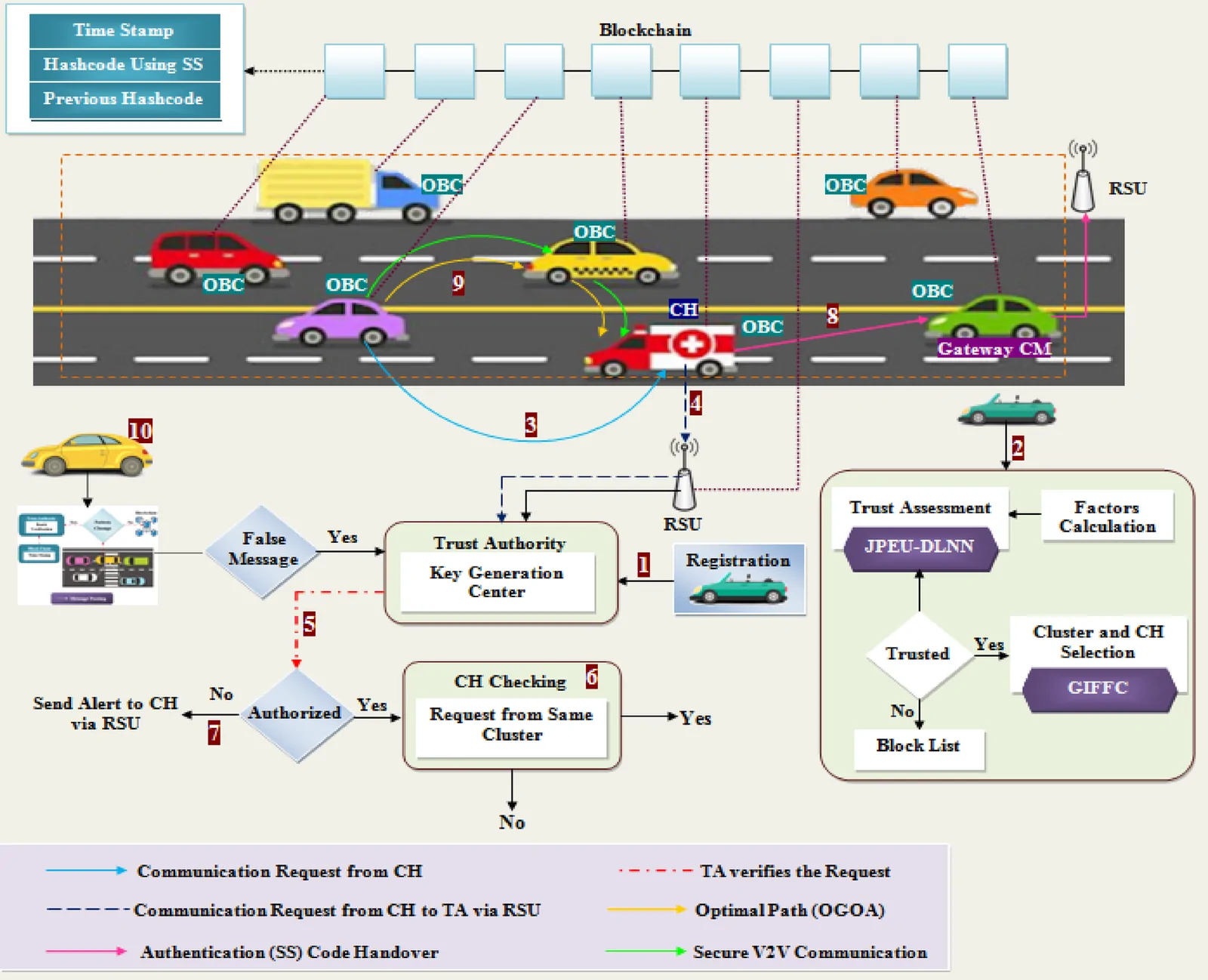
System architecture of the proposed blockchain-enabled trust-aware authentication framework showing vehicle, cluster head, RSU, trust evaluation, and blockchain interaction.

The proposed framework consists of four major components: (1) blockchain-based credential storage, (2) JPEU-DLNN-based trust assessment, (3) GIFFC-based cluster formation, and (4) DGOA-based optimal path selection. The overall workflow ensures secure authentication, mobility-aware handover, and resilient communication in VANET environments. In VANETs, security and latency are the major concerns during information sharing. Security of the network can be improved by granting access only to authenticated vehicles and utilizing a secure platform to authorize the vehicles. Hence, a novel blockchain-based authorization framework in VANET using the LECC with a novel GIFFC scheme is proposed. The block representation of the proposed framework is given in Fig. 1,

### Architecture overview

The proposed framework consists of five main components: Vehicles (OBUs), Cluster Heads (CHs), Roadside Units (RSUs), Trust Evaluation Module, and a Permissioned Blockchain Network.

Vehicles generate cryptographic credentials using LECC and initiate authentication requests when joining clusters or during RSU handover. The JPEU-DLNN model evaluates vehicle behavior to compute dynamic trust scores and detect malicious nodes. The GIFFC algorithm forms stable clusters, while DGOA optimizes routing paths to reduce delay. Authentication results and trust updates are securely recorded in the blockchain to ensure decentralization and immutability.

Figure 1 illustrates the overall architecture of the proposed blockchain-enabled trust-aware authentication framework. The vehicles equipped with OBUs communicate with cluster heads (CHs), which forward authentication requests to Roadside Units (RSUs). The trust evaluation module analyzes vehicle behavior and generates trust scores. Cryptographic validation using LECC is performed before authentication records are appended to the permissioned blockchain. The arrows in the figure represent the authentication request flow, trust verification process, and blockchain update mechanism.

Although Roadside Units (RSUs) are physically static infrastructure components, employing a centralized authentication server introduces single-point failure, scalability limitations, and trust dependency issues in VANET environments. The proposed framework utilizes a distributed permissioned blockchain among RSUs to decentralize authentication control and eliminate reliance on a single trusted authority.

In this architecture, multiple RSUs act as blockchain validator nodes that collectively verify and record authentication events. This distributed validation mechanism ensures:

- Fault tolerance in case of RSU failure.
- Tamper-resistant storage of authentication records.
- Load sharing across infrastructure nodes.
- Resistance to insider manipulation attacks.

Therefore, despite the static deployment of RSUs, the logical authentication process remains decentralized, enhancing system robustness and trust management in dynamic vehicular communication environments.

#### Role of blockchain in DL-VAuth framework

In the proposed VANET authentication architecture, blockchain functions as a decentralized trust management layer that securely maintains vehicle identity credentials, session token hashes, and authentication records without relying on a centralized Trusted Authority (TA). The Road Side Units (RSUs) act as blockchain nodes that validate and append authentication transactions generated during the vehicle-to-infrastructure communication process.

Whenever a vehicle initiates authentication, its identity-bound token is verified through smart-contract-based consensus maintained across RSU nodes. The blockchain ledger stores only hashed authentication parameters and session key validation tags, ensuring immutability, traceability, and resistance against modification and insider attacks. This integration prevents forged identity registration and enables distributed session verification among participating entities.

#### System and security assumptions

The proposed blockchain-enabled trust-aware authentication framework is designed under the following assumptions:

1. A trusted authority exists during the initial registration phase to securely register vehicles and RSUs before deployment.
2. The blockchain operates in a permissioned environment where RSUs and selected cluster heads validate and store authentication records immutably.
3. Vehicles are equipped with tamper-resistant Onboard Units (OBUs) that securely store private keys generated using Log-based Edwards Curve Cryptography (LECC).
4. Adversaries can eavesdrop, replay, modify, or inject messages over insecure wireless channels but cannot compromise blockchain consensus or solve the Elliptic Curve Discrete Logarithm Problem (ECDLP).
5. Vehicles are highly mobile, and authentication continuity during cluster transitions and RSU handover is supported through blockchain-based verification.
6. Vehicles and RSUs are loosely time-synchronized to prevent replay attacks.

The proposed VANET environment consists of heterogeneous nodes with varying computational and communication capabilities. Vehicles equipped with On-Board Units (OBUs) are resource-constrained and perform lightweight cryptographic operations. Cluster Heads (CHs) possess moderate processing capability to manage intra-cluster authentication requests. Roadside Units (RSUs) are infrastructure-supported nodes with higher computational power and storage capacity, enabling blockchain validation and authentication record maintenance.

### Authentication entities and their roles

The proposed framework involves four primary entities participating in the authentication process:

1. Vehicle (OBU): Each vehicle initiates authentication requests when joining a cluster or during RSU handover. Vehicles authenticate themselves to Cluster Heads (CHs) and RSUs before secure communication is established.
2. Cluster Head (CH): The CH verifies vehicle authentication requests within the cluster and forwards validated information to the RSU for blockchain recording. The CH also participates in trust evaluation.
3. Roadside Unit (RSU): The RSU performs final authentication validation and acts as a blockchain validator node. It stores authentication records immutably in the permissioned blockchain.
4. Blockchain Network: The blockchain maintains tamper-resistant authentication logs and ensures decentralized verification without relying on a centralized authority.

**Figure:**
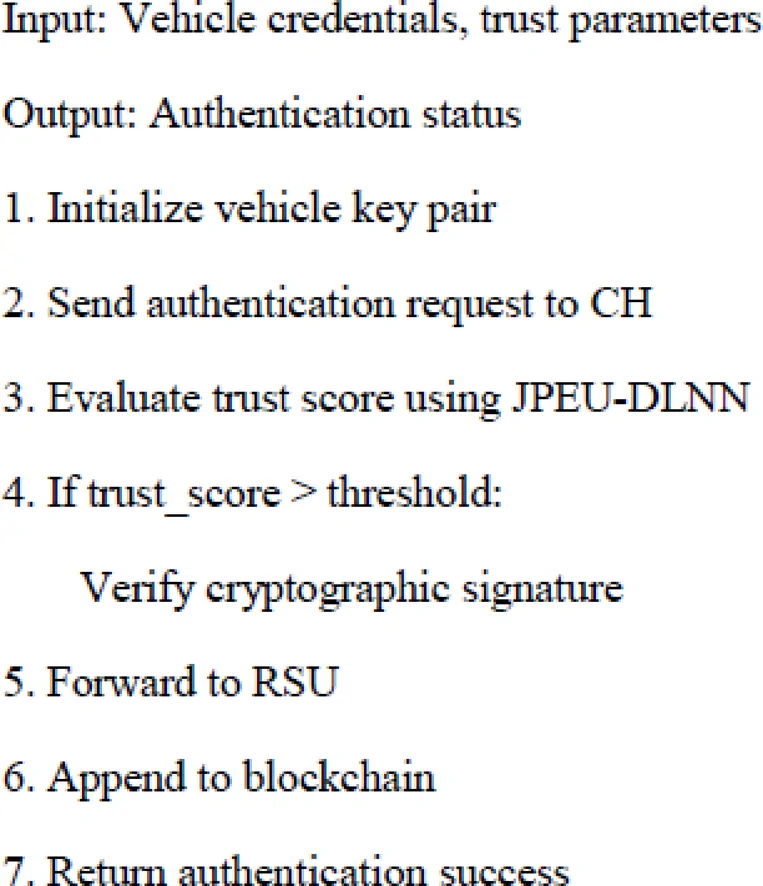

#### Initialization

At first, the VANET network model is initialized. In VANET, the vehicles (nodes) with OBU are presented in a random manner. The mathematical model of VANET $\:\left(V\right)$is depicted as,

1 $$\:V=\left\{{v}_{1},{v}_{2},....,{v}_{n}\right\}or{v}_{node}$$

Where, $\:{v}_{n}$depicts the $\:{n}^{th}$Vehicle Node (VN) in the VANET.

#### Authentication

For authentication, initially, the vehicles and the RSUs are registered with the Trusted Authority (TA).The RSUs perform registration with their factors, such as capacity, signal strength, and so on with the TA. For registration, the Vehicle Users (VUs) register their credential information and OBU details. During registration, the keys and hashcodes are generated for each vehicle node, which was needed for authentication. The authenticated VN’s information is stored in the blockchain. Table 3.

**Table 3:** Notation and symbol description.

Symbol	Description
Vi	Vehicle i
RSUj	Roadside Unit j
SK	Session Key
RID	Real Identity
PID	Pseudo Identity
r1, r2	Random Numbers
H(.)	Secure Hash Function
TID	Temporary Identity
TS	Timestamp

### Key generation

Each vehicle generates a lightweight elliptic curve (LECC) public-private key pair during initialization. The public key is registered with the RSU and recorded in the permissioned blockchain.

### Key update mechanism

To ensure forward secrecy and reduce key compromise risk, session keys are dynamically generated for each authentication session using:

- Vehicle private key.
- RSU public key.
- Random nonce and timestamp.

Session keys are valid only for a limited time interval and are automatically refreshed during:

- Cluster change.
- RSU handover.
- Periodic re-authentication.

The Edward Curve Cryptography (ECC) is considered as more secure by selecting the base point from the closed formation of the curve with addition and doubling law. Still, the ECC has complexity problems for larger numbers. To solve this problem, the Logarithmic calculation is used to reduce the complexity of ECC. The key generation with LECC is given as,

In the LECC algorithm, four points are selected from the Edward curve equation,

2 $$\:{u}^{2}+{v}^{2}=1+e.{u}^{2}{v}^{2}$$

Where, * u,v* depicts * u*and* v* axes respectively. A scalar value is given by $\:e\in\:I$, where * I*depicts the finite field over the Edward curve. From the curve, two points $\:\left({i}_{1},{i}_{2}\right)$are considered the private keys. With the private keys, the public keys are generated as,

3 $$\:{p}_{1}=loglog\:\left(R\right)\:.{i}_{1}$$

4 $$\:{p}_{2}=loglog\:\left(R\right)\:.{i}_{2}$$

Where, $\:{p}_{1},{p}_{2}$signifies the generated public keys at the TA and VUs side, and* R*depicts the basepoint obtained from the Edward curve.

### Hashcode generation

Meanwhile, in the proposed model, the hashcode for authentication is also generated by the Split Skein (SS) hashing algorithm and stored in the blockchain. The conventional skein hashing is made by the three-fish cipher approach, which provides secure hashcode. But, the skein provides a very lengthy code for each round; so, it makes more complex code. To overcome this problem, the blocks of a few words are considered as code and a final hash value is the combination of selected code words. The SS hashing is depicted as follows,

The input to the SS hashing is the credential information of VU and OBU details of VN. The information detail plain text is denoted as$\:\left(A\right)$. The basic component of SS hashing is the three-fish block cipher function, which is performed inside each Unique Block Iteration (UBI) chaining mode.

#### Threefish

In the threefish technique, a key length of * l* bytes are used and the block length (plain text) is equal to the * l*. The tweak value of 128 bits is irrespective of the block length. Depending upon the blocksize, several rounds are performed using arithmetic operators. The threefish can be denoted as $\:tf\left(I,A,\lambda\:\right)$, where * I* denotes the current tweak value and $\:\lambda\:$ indicates the key value. A single threefish round is a set of * l/2* mix operations and a single permute operation. In the proposed model, to reduce the number of rounds, the block is split as,

5 $$\:A=\left\{{R}_{1},{R}_{2},.....,{R}_{be}\right\}or{R}_{sp},sp=\mathrm{1,2},..,be$$

Where, $\:{R}_{be}$ depicts the end block $\:\left(be\right)$, * sp* depicts the split blocks. Then, the split blocks are divided with 64 as three-fish, which operates on a 64-bit word, which can be formulated as,

6 $$\:{\vartheta\:}_{A}=\frac{{R}_{sp}}{64}$$

Here, $\:{\vartheta\:}_{A}$ denotes the number of 64-bit words in the input message block. Within threefish, three operations take place: mix, permute, and sub-key addition.

#### Threefish subkey generation

Threefish generates $\:\frac{{\vartheta\:}_{rnd}}{4}+1$ subkeys from the cipher key $\:\left(\lambda\:\right)$, where, $\:{\vartheta\:}_{rnd}$ depicts the number of rounds. Along with $\:\lambda\:$, a 64-bit constant value $\:\left(\varsigma\:\right)$ and * I*, the subkeys $\:\left({\lambda\:}_{0},{\lambda\:}_{1},....,{\lambda\:}_{l-1}\right)$ are generated. Prior to sub-key generation, the two 64-bit words of tweak value $\:\left(t{t}_{0},t{t}_{1}\right)$ is extended to a word $\:t{t}_{2}$ and a new key $\:\left({\lambda\:}_{l}\right)$ as,

7 $$\:t{t}_{2}=t{t}_{0}\oplus\:t{t}_{1}$$

8 $$\:{\lambda\:}_{l}=\varsigma\:\oplus\:{\lambda\:}_{0}\oplus\:{\lambda\:}_{1}\oplus\:......\oplus\:{\lambda\:}_{l-1}$$

In every round, the subkeys are defined as,9

Where, $\:0\le\:jj\le\:{\vartheta\:}_{A}-1$and$\:0\le\:sk\le\:\frac{{\vartheta\:}_{rnd}}{4}$, $\:\oplus\:$ represents the bitwise XOR operation, and $\:\varXi\:$ signifies the addition modulo 2^64^.

#### Mix and permutations

In threefish, the mix and permutations process is performed in each step of encryption. The encryption is done by adding subkeys to the plain words. The mix operation on 64-bit words is given as,

10 $$\:m{x}_{sk,0}=e{n}_{sk,0}\varXi\:e{n}_{sk,1}$$

11 $$\:m{x}_{s,1}=\left(e{n}_{sk,1}<<<J\right)\oplus\:m{x}_{sk,0}$$

Where, * <<<J* depicts the bit rotated to left in * J* times, * J* is a constant value. After the mixing operations $\:\left(m{x}_{sk,0},m{x}_{s,1}\right)$, the mixed outputs are permuted and produce the encrypted message.

#### UBI

The skein contains three UBIs for hashing: configuration UBI, message UBI, and Output UBI. The skein hashing can be modelled as,

12 $$\:SH=\upsilon\:\left({\sigma\:}_{jj-1},O,{I}_{sk}\right)$$

Where, $\:\upsilon\:({\sigma\:}_{jj-1)}$depicts the UBI function, $\:O,{I}_{sk}$depicts the message of arbitrary length and tweak value, and$\:\sigma\:$depicts the chaining value.

Initially, a message $\:{R}_{be}$ is given to configuration UBI, which produces a chain value process in configuration UBI, which is represented as, $\:{\sigma\:}_{0}=\upsilon\:\left({\lambda\:}^{{\prime\:}},\varsigma\:,{I}_{cnfg}\right)$. This $\:{\sigma\:}_{0}$ is given to the message UBI, where multiple threefish block ciphers are used to process the input message$\:{R}_{be}$. The message UBI process is denoted as, $\:\sigma\:=\upsilon\:\left({\sigma\:}_{0},{R}_{be},{I}_{mesg}\right)$. Then, $\:\sigma\:$ is given to output UBI and generates $\:{\vartheta\:}_{out}$ number of the final hash value. The final hash function for $\:{\vartheta\:}_{out}$ bits is formulated as,

13 $$\:{H}_{be}=\upsilon\:\left(\sigma\:,0,{I}_{out}{2}^{120}\right)\left|\right|\upsilon\:\left(\sigma\:,1,{I}_{out}{2}^{120}\right)\left|\right|\upsilon\:\left(\sigma\:,2,{I}_{out}{2}^{120}\right)..$$

Where $\:{H}_{be}$depicts the hashed output for the input $\:{R}_{be}$. Similarly, the hash value for all the blocks is evaluated and the sum of the hashes is taken as the required hashed output.

14 $$\:{H}_{U}={\sum\:}_{sp=1}^{be}{H}_{sp}$$

The final hash code for the VN $\:\left(U\right)$ is depicted as $\:{H}_{U}$. This hashcode $\:{H}_{U}$ is stored in the block of the blockchain.

For the VUs and RSA, the TA gives the dummy ID for further data communication; thus, the dummy values are also stored in the blockchain.

### Factor calculation

After the block creation in the blockchain, the network deployment is performed, where the pre-trails of message passing are performed to obtain factors. The network deployment is calculated, which is given as,

15 $$\:F=\left\{{f}_{1},{f}_{2},{f}_{3},{f}_{4},{f}_{5},{f}_{6}\right\}or{f}_{x},x=1,....,6$$

Where, * F* depicts the factor set, $\:{f}_{1}$ depicts the data forwarding rate, $\:{f}_{2}$ indicates the consistency, $\:{f}_{3},\:{f}_{4}$ signifies the time and packet loss, and $\:{f}_{5},\:{f}_{6}$ represents the sending rate and message rating.

### Trust assessment

**Fig. 2:**
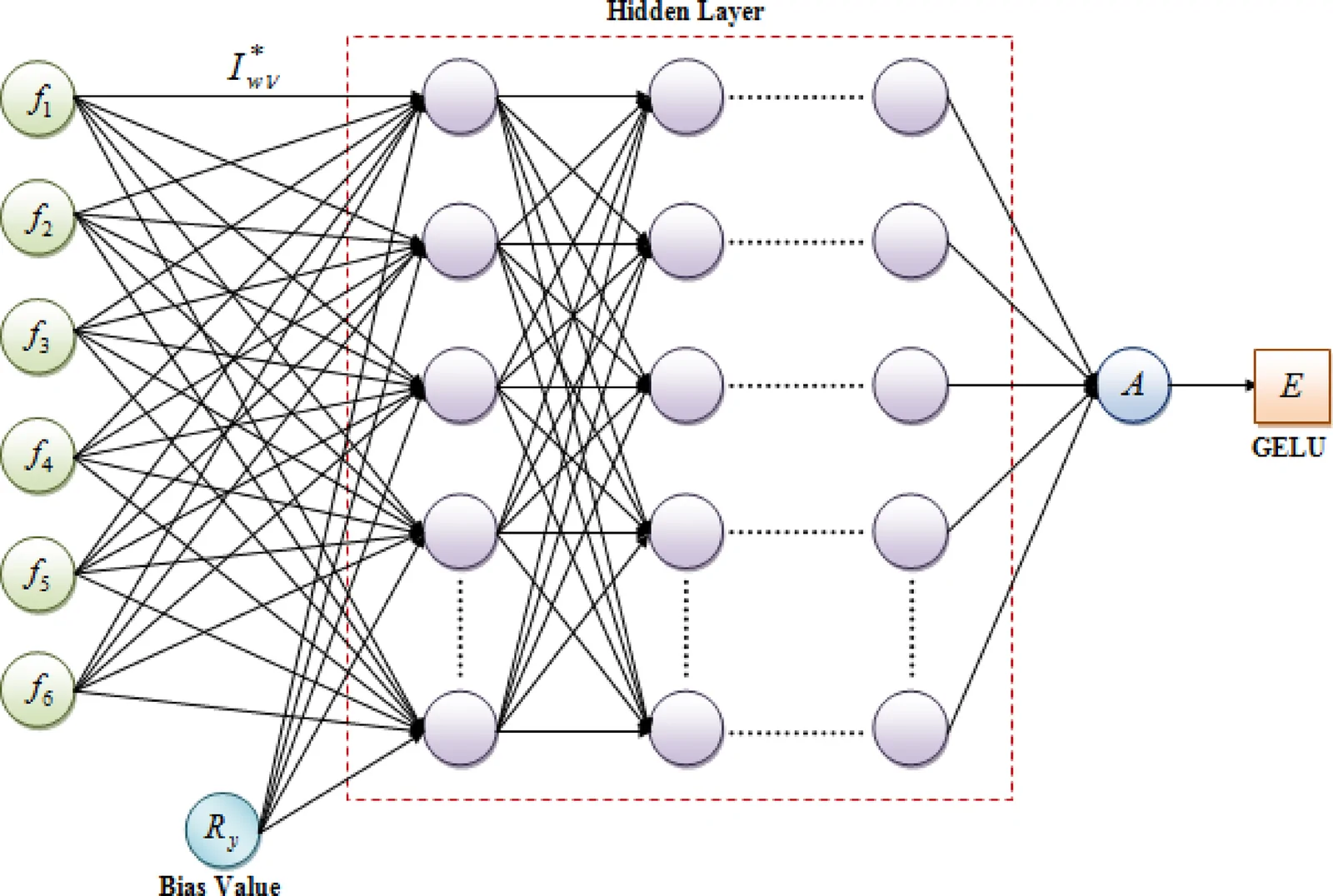
Proposed JPEU-DLNN.

The obtained factors $\:\left\{{f}_{x}\right\}$ are then used for trust assessment. In the proposed framework, the Joint Probability and Error Unit-based Deep Learning Neural Network (JPEU-DLNN) algorithm is proposed for trust assessment. Here, the Deep Learning Neural Network (DLNN) is considered for trust assessment since the DLNN gives more accurate results. Yet, the DLNN has the vanishing gradient problem and black box problem. To solve those problems, this research methodology calculates the joint probability of input, weight, and bias values for the individual neurons. During the calculation, the combination with better probability is selected as the weight and bias value for all the neurons. Also, the error value is calculated using the Gaussian Error Linear Unit (GELU), which makes the system faster. The working procedure of JPEU-DLNN is given in Fig. 2,

#### Input layer

In JPEU-DLNN, the input is the obtained factors $\:\left\{{f}_{x}\right\}$. In the input layer, the factors are prepared to be processed by the further layers.

#### Weight and Bias values

For each input neuron, a weight value and bias value are assigned with the joint probability $\:\left({P}_{x}\right)$ model. For that, the weight $\:\left({I}_{wv},wv=\mathrm{1,2},....,5\right)$ and bias $\:\left(R\right)$ values,

16 $$\:{P}_{x}=\frac{expexp\:\left(-\rho\:\left({f}_{x},{I}_{wv},R\right)\right)\:}{{\sum\:}_{x=1}^{5}expexp\:\left(-\rho\:\left({f}_{x}\right)\right)\:}$$

Here, $\:\rho\:\left({f}_{x},{I}_{wv},R\right)$ depicts the probability function. Likewise, the probability for all hidden units is estimated and $\:\left({P}_{x}\right)\:$is selected as weight and bias values for all the input neurons. The selected weight and bias value are denoted as $\:\left({I}_{wv}^{*},{R}_{y}\right)$.

#### Hidden layer

In the hidden layer, each neuron sums up the input neuron value with the corresponding weight and bias values to produce the significant features. The hidden layer $\:\left(H{i}_{x}\right)$ computation is given as,

17 $$\:H{i}_{x}={\sum\:}_{x=1}^{5}{I}_{wv}^{\mathrm{*}}.{f}_{x}+{R}_{y}$$

Where, $\:H{i}_{x}$ depicts the hidden neuron process.

#### Output layer

The output layer sum up the hidden layer output with its corresponding weights and bias values to give the assessment value. The output neuron process is given as,

18 $$\:A={\sum\:}_{x=1}^{5}H{i}_{x}.{I}_{wv}^{\mathrm{*}}+{R}_{y}$$

Here, * A* depicts the assessment output, and $\:{R}_{y}$ denotes the bias value of the hidden layers.

#### Error estimation

After the output is obtained, the error in the output is estimated to evaluate whether the obtained output meets the target output value. In the proposed JPEU-DLNN, the error $\:\left(E\right)$ is estimated with the GELU activation function.

19 $$\:E=\frac{1}{2}A.\left(1+\varTheta\:\left(\frac{A}{\sqrt{2}}\right)\right)$$

Here, $\:\varTheta\:\left(\frac{A}{\sqrt{2}}\right)$ represents the error function, if * E=threshold*, the assessment result is considered, or else the weight values are updated and the output is predicted. Thus, by doing this, the trusted and untrusted users in the VANET are predicted. After the trusted and non-trusted users are classified, the non-trusted users are blocked in the VANET for information sharing and stored in the blocklist. The trusted VUs are selected for further processing and the selected trusted VUs are mathematically represented as,

20 $$\:T=\left\{{t}_{1},{t}_{2},....,{t}_{\alpha\:}\right\}or{t}_{vu},vu=\mathrm{1,2},....,\alpha\:$$

Where, * T* depicts the VANET and $\:{t}_{\alpha\:}$ indicates the $\:{\alpha\:}^{th}$ trusted VU. The pseudocode of the proposed JPEU-DLNN is given as follows,

**Figure:**
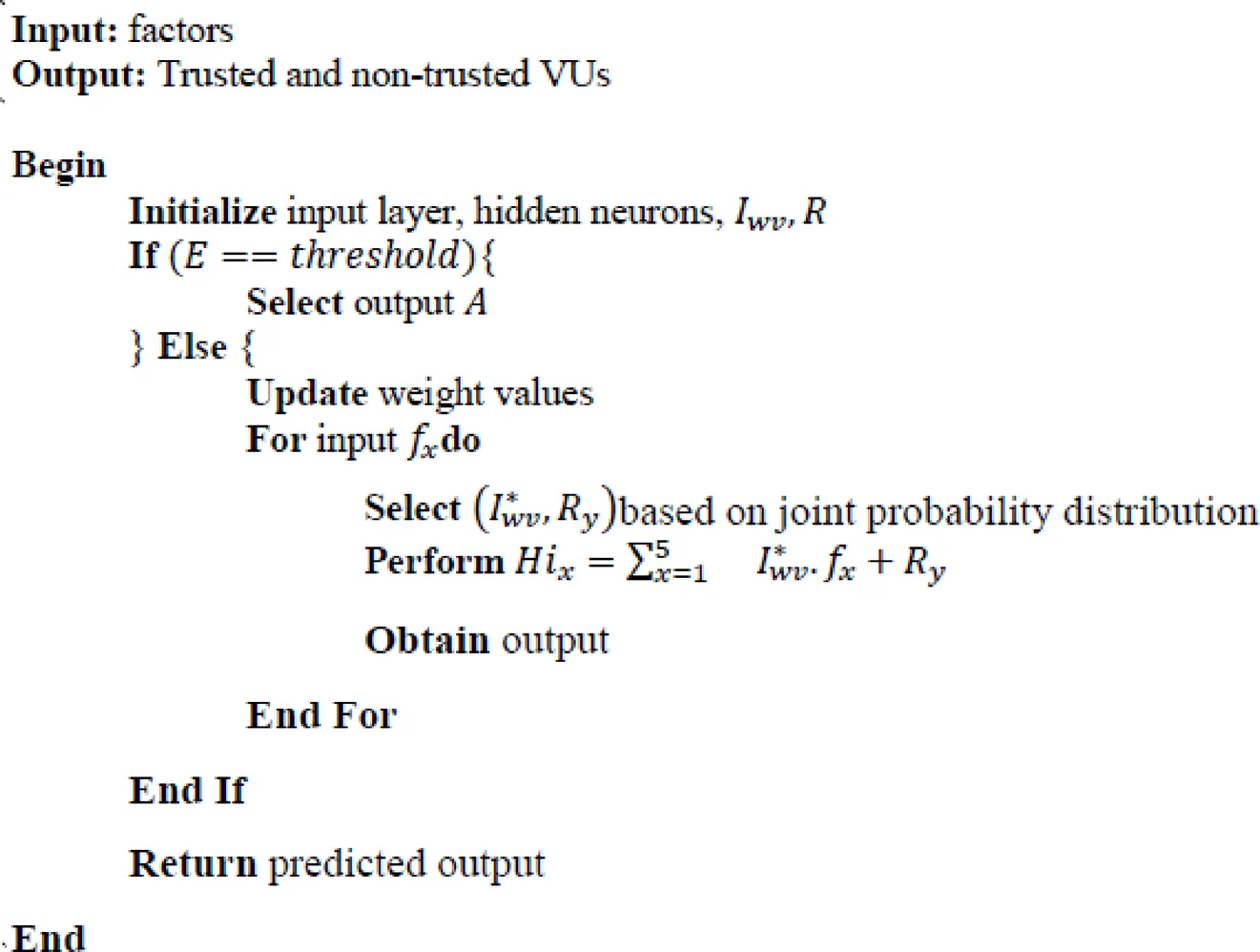

### Clustering and cluster head (CH) selection

After the trusted and non-trusted users are classified, the non-trusted users are blocked in the VANET for information sharing and are stored in the blocklist. A cluster is formed by the trusted Vehicles from which the cluster head is selected which acts as the gateway node to share information with other users, which reduces the communication time. In the proposed model, a primary CH and two secondary CHs are selected by using the Gini Indexed Farthest First Clustering (GIFFC) algorithm based on the data forwarding rate, consistency factor, time factor, packet loss factor, and sending rate factor. The Farthest First Clustering (FFC) is considered in the proposed model due to its efficient clustering for a large number of Vehicles. Yet, in FFC, the first point is randomly chosen and then the maximum distance value from the selected first point is taken as the farthest cluster centroids, which ignited cluster outlier problems and inaccurate clustering. To solve this, Gini Index (GI) value for the input values is calculated, then a higher index value is chosen as the first centroid and the maximum distance from the first centroid is chosen as the farthest centroid point. Thus, the GIFFC clustering procedure is as follows,

In the GIFFC algorithm, the first cluster center is selected by using the Gini index$\:\left(G\right)$,

21 $$\:{G}_{vu}=1-{\sum\:}_{vu=1}^{\alpha\:}{\nabla\:}^{2}\left({t}_{vu}\right)$$

Where, $\:\nabla\:\left({t}_{vu}\right)$ depicts the proportion of the vehicle node $\:\left(vu\right)$. After the Gini index is calculated for all vehicles, the VU with the maximum Gini index is chosen as the first cluster center. Here, the first cluster center, which has the highest Gini Index, is denoted as the cluster center $\:\left(ch\right)$.

After the cluster center is identified, the distance (similarity) between the $\:\left(ch\right)$ and the remaining vehicles is estimated. The distance $\:\left(D\right)$ calculation is mathematically depicted as,

22 $$\:D={\sum\:}_{vu=1}^{\alpha\:}{\Vert\:{G}_{vu}-ch\Vert\:}^{2}$$

The $\:\left(vu\right)$ with the farthest distances are selected as the other cluster centers and the $\:\left(vu\right)$ with the nearest distance is taken as cluster members (CMs). Thus, the cluster is given as,

23 $$\:C=\left\{c{m}_{1},c{m}_{2},.....,c{m}_{cc}\right\},orc{m}_{uu},uu=\mathrm{1,2},...,cc$$

Here, * C*represents the cluster of vehicles and $\:c{m}_{cc}$ indicates the $\:c{c}^{th}$ cluster member. From the cluster $\:\left(C\right)$, three CHs are selected based on the trusting $\:\left(trust\right)$, message rating $\:\left(mr\right)$ of each node, residual strength $\:\left(Rs\right)$, location$\:\left(loc\right)$, speed $\:\left(spd\right)$, velocity $\:\left(vel\right)$, and equipment of vehicles $\:\left(eov\right)$ as,

24 $$\:CH\leftarrow\:\left(trust,mr,Rs,loc,spd,vel,eov|c{m}_{uu}\right)\:$$

The CM who has the maximum of * trust,mr,Rs,loc,spd,vel,eov* is selected as the primary CH and the other two act as secondary CHs. The GIFFC pseudocode is stated as follows,

**Figure:**
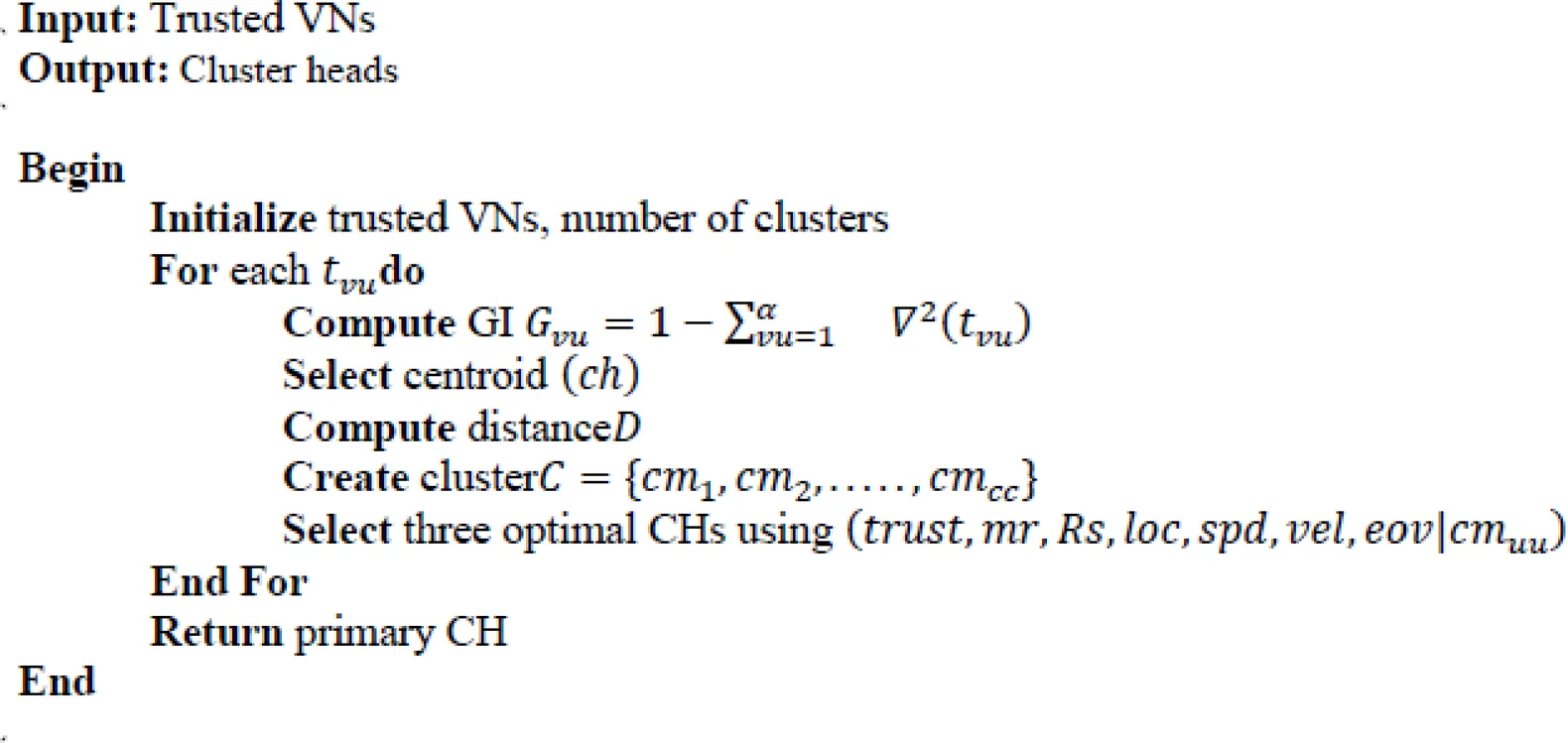

The proposed framework is designed as an integrated system in which all components operate cohesively. The trust evaluation module plays a critical role in identifying reliable nodes, which are subsequently selected as cluster heads to facilitate efficient communication and coordination within the network.Furthermore, blockchain technology ensures secure authentication and data integrity by maintaining tamper-proof records of all transactions. These authenticated records are utilized to support secure routing and path optimization, thereby improving overall network performance. This integration of trust evaluation, clustering, and blockchain-based authentication eliminates isolated processing of modules and ensures a unified and efficient system architecture suitable for dynamic VANET environments.

### Verification process

When a VN $\:\aleph\:$, in the cluster wants to get information, it sends a request to the CH$\:\left(c{h}_{primary}\right)$ of the corresponding cluster. The Cluster Head (CH) initiates a communication request to the Trusted Authority (TA) through the Roadside Unit (RSU) for verification. Upon receiving the request, the TA evaluates whether the Vehicular Node (VN) is authorized or not. If the VN is identified as unauthorized, the TA issues an alert message back to the CH via the corresponding RSU.

- If the $\:\aleph\:$ is authorized, the CH checks whether the $\:\aleph\:$is present in the same cluster or not.
- If $\:\aleph\:$ is present in the same cluster, an optimal route is selected, and data communication takes place. Else if the $\:\aleph\:$ is not present in the same cluster, the authentication code handover takes place.

### Optimal path selection

For reliable communication, the optimal path is selected. The proposed framework utilizes Directional Gannet Optimization Algorithm (DGOA). The GOA algorithm provides better performance but it finds the feasible solution based on the weight and speed of the gannet. This process may provide less effort to find a feasible solution so that this research methodology incorporates the direction of the search within the finding of a feasible solution.

#### Initialization

In DGOA, the input is the initial population, which is considered as the V2V hops in the corresponding cluster of $\:\aleph\:$. The position of the gannet within the dimension * z* is given as,

25 $$\:{g}_{b,z}=r{a}_{1}\left(u{b}_{z}-l{b}_{z}\right)+lb$$

Where, $\:{g}_{b,z}$ depicts the position of the gannet * b*, in the dimension * z*, * lb,ub* depicts the lower and upper limit of the dimension * z*, and $\:r{a}_{1}$ indicates a random number. Thus, by calculating this, the position of all the gannets is obtained, which can be represented in the matrix as,

26 $$\:G=\left[{g}_{\mathrm{1,1}}{g}_{\mathrm{1,2}}\cdots\:{g}_{1,D}\:{g}_{\mathrm{2,1}}{g}_{\mathrm{2,2}}\cdots\:{g}_{2,D}\:\vdots\vdots\ddots\:\vdots\:{g}_{\zeta\:,1}{d}_{\zeta\:,2}\cdots\:{g}_{\zeta\:,D}\:\right]or\left[{g}_{b,z}\right],b=\mathrm{1,2},...,\zeta\:,z=\mathrm{1,2},....,D$$

Where, the gannet population is represented as * G*, * D,z* represents the size of the dimension and population. Also, a gannet memory matrix * GM* is generated, in which the values of * G* are assigned initially. But, whenever the position of the gannet changes, the fitness function of the current position in the memory matrix is compared, and the best position is updated in the * GM*. Here, the fitness function $\:\left(Fi\right)$ is represented as,

27 $$\:Fi\leftarrow\:\langle\left(Rs,Re\left(\right)\left(Dst\right)\:\right)\:\langle\rangle\rangle$$

Where, $\:Rs,D\:st$ depicts the residual signal strength, residual energy, and distance respectively.

#### Exploration phase

The Gannet search for prey in the water from the air. If a prey is found, the dive pattern is adjusted as a V-shaped or U-shaped dive to attack the prey, which can be mathematically denoted as,

$$\:u-s=2*coscos\:\left(2*\pi\:*r{a}_{2}\right)\:*ts$$

28 $$\:v-s=2\mathrm{*}\kappa\:\left(2\mathrm{*}\pi\:\mathrm{*}r{a}_{3}\right)\mathrm{*}ts$$

29 $$\:ts=1-\frac{Iterarion}{Iteratio{n}_{max}}$$

30 $$\:\kappa\:\left(g\right)=\{-\frac{1}{\pi\:}\mathrm{*}g+1ifg\in\:\left(0,\pi\:\right)\:\frac{1}{\pi\:}\mathrm{*}g-1ifg\in\:\left(\pi\:,2\pi\:\right)\:$$

Here, $\:Iteration,Iteratio{n}_{max}$ depicts the current iteration, * ts* denotes the time step, and * u-s,v-s* signifies the U-shape and V-shape dive. The gannet chooses one of the dive procedures to update its position based on the random number $\:\psi\:$ as,

31 $$\:G{M}_{b}\left(ts+1\right)=\{{G}_{b}\left(ts\right)+{\beta\:}_{1}+{\beta\:}_{2}if\left(\psi\:\ge\:0.5\right)\:{G}_{b}\left(ts\right)+{\gamma\:}_{1}+{\gamma\:}_{2}if\left(\psi\:<0.5\right)\:$$

$$\:{\beta\:}_{2}=Z*\left({g}_{b}\left(ts\right)-{g}_{ra}\left(ts\right)\right)$$

32 $$\:{\gamma\:}_{2}=Y\mathrm{*}\left({g}_{b}\left(ts\right)-{\stackrel{-}{g}}_{b}\left(ts\right)\right)$$

$$\:Z=\left(2*r{a}_{4}-1\right)*u-s$$

33 $$\:Y=\left(2\mathrm{*}r{a}_{5}-1\right)\mathrm{*}vs$$

Where, $\:r{a}_{4},\:r{a}_{5}$ indicates random numbers and $\:{\beta\:}_{1},{\gamma\:}_{1}$ depicts the random number between $\:\left(-us,us\right)$ and $\:\left(-vs,vs\right)$respectively. $g_{ra}$ denotes a random gannet. $\:{\stackrel{-}{g}}_{b}\left(ts\right)$ depicts the mean position of an individual gannet, which can be evaluated using,

34 $$\:{\stackrel{-}{g}}_{b}\left(ts\right)=\frac{1}{\zeta\:}{\sum\:}_{b=1}^{\zeta\:}{g}_{b}\left(ts\right)$$

#### Exploitation phase

As the gannet performs one of the dive mechanisms, the fish tries to escape through a sudden turning movement. The capturing ability $\:\left(CA\right)$ of the gannet is based on sufficient energy, which can be defined as,

35 $$\:CA=\frac{1}{W\mathrm{*}\left(t{s}_{2}\right)}$$

$$\:t{s}_{2}=1+\frac{Iteration}{Iteratio{n}_{max}}W=\frac{{\mu\:}^{2}*\varpi\:*d\overrightarrow{d}}{\varGamma\:}$$

36 $$\:\varGamma\:=0.2+\left(2-0.2\right)\mathrm{*}r{a}_{6}$$

Where, $\:r{a}_{6}$ depicts a random number, $\:\mu\:,\varpi\:$ depicts the velocity and weight of the gannet, $\:\varGamma\:$ signifies specific velocity, * W*denotes the energy of the gannet, and $\:t{s}_{2}$ depicts the second time step. In the proposed direction, $\:d\overrightarrow{d}$ gannet has introduced fast convergence of GOA.

If the * CA* of gannet is within the range of catchable prey, the position is updated as,

37 $$\:G{M}_{b}\left(ts+1\right)=\{ts\mathrm{*}\varDelta\:\mathrm{*}\left({g}_{b}\left(ts\right)-{g}^{{\bullet\:}}\left(ts\right)+{g}_{b}\left(ts\right)\right)if\left(CA\ge\:cc\right)\:{g}^{{\bullet\:}}\left(ts\right)-\left({g}_{b}\left(ts\right)-{g}^{{\bullet\:}}\left(ts\right)+{g}_{b}\left(ts\right)\right)\mathrm{*}\chi\:\mathrm{*}tselse\:$$

$$\:\varDelta\:=CA*\left|{g}_{b}\left(ts\right)-{g}^{\bullet\:}\left(ts\right)\right|$$

38 $$\:\chi\:=lev\left(D\right)$$

If $\:\left(CA\ge\:cc\right)$, the gannet attacks the prey by updating the position, or else the gannet is unable to catch the fish and performs Levy movement in search of the next prey. Here, * cc=0.2* depicts a random number, $\:\varDelta\:$ depicts the displacement, $\:{g}^{\bullet\:}\left(ts\right)$ depicts the best position, and $\:\chi\:$ indicates the movement of the gannet to the next prey. $\:lev\left(D\right)$ signifies the levy flight function, which is formulated as,

39 $$\:lev\left(D\right)=0.01\left(\frac{{\wp\:}\times\:\delta\:}{{\left|\varOmega\:\right|}^{\frac{1}{{\overline\lambda}}}}\right)$$

Where, $\:\wp\:,\delta\:$ are random values, $\overline\lambda=1.5$ is predetermined constant. Thus, by updating $\:G{M}_{b}\left(ts+1\right)$ and evaluating the fitness function, the best solutions (best hops) are obtained, which creates an optimal path for data transmission.

### Authentication code handover

Meanwhile, if the $\:\aleph\:$does not present in the same cluster as CH, $\:\aleph\:$ is likely present in the nearby cluster. Thus, to be authenticated by the nearby RSU, the authentication code of $\:\aleph\:$ generated by the SS algorithm is passed to the nearby RSU. During the authentication handover, a CM acts as the gateway node, which collects the authentication code from the CH and handover to the nearby RSU. Then, the $\:\aleph\:$ gets authenticated in the nearby cluster and performs data sharing by determining the optimal path using DGOA.

### Secure data communication

After the optimal path is selected, secure communication takes place among the CMs present in the optimal path using the LECC algorithm in which the required data is encrypted from the one VN and shared to the adjacent VN where decryption takes place. The LECC encryption and decryption are stated as follows,

#### Encryption

During encryption, the information need to be transferred to the receiver VN$\:\left({v}_{recieve}\right)$ is converted into cipher text as,

$$\:C{i}_{1}=rn.R$$

40 $$\:C{i}_{2}=\tau\:+{p}_{2}.rn$$

Where, $\:\left(C{i}_{1},C{i}_{2}\right)$ indicates the encrypted cipher texts that are to be sent from $\:{v}_{node}$ to the $\:{v}_{receive}$, $\:\tau\:$ signifies the information to be shared and * rn* denotes the random number.

#### Decryption

The $\:\left(C{i}_{1},C{i}_{2}\right)$ are decrypted to plain text in the $\:V{N}_{receive}$ as,

41 $$\:Inf=C{i}_{2}-{i}_{2}.C{i}_{1}$$

Here, * Inf* depicts the decrypted information, and $\:{i}_{2}$ is the $\:{v}_{receive}$ private key. Thus, by performing LECC, data communication between the vehicle nodes can be secured.

### Batch verification

**Fig. 3:**
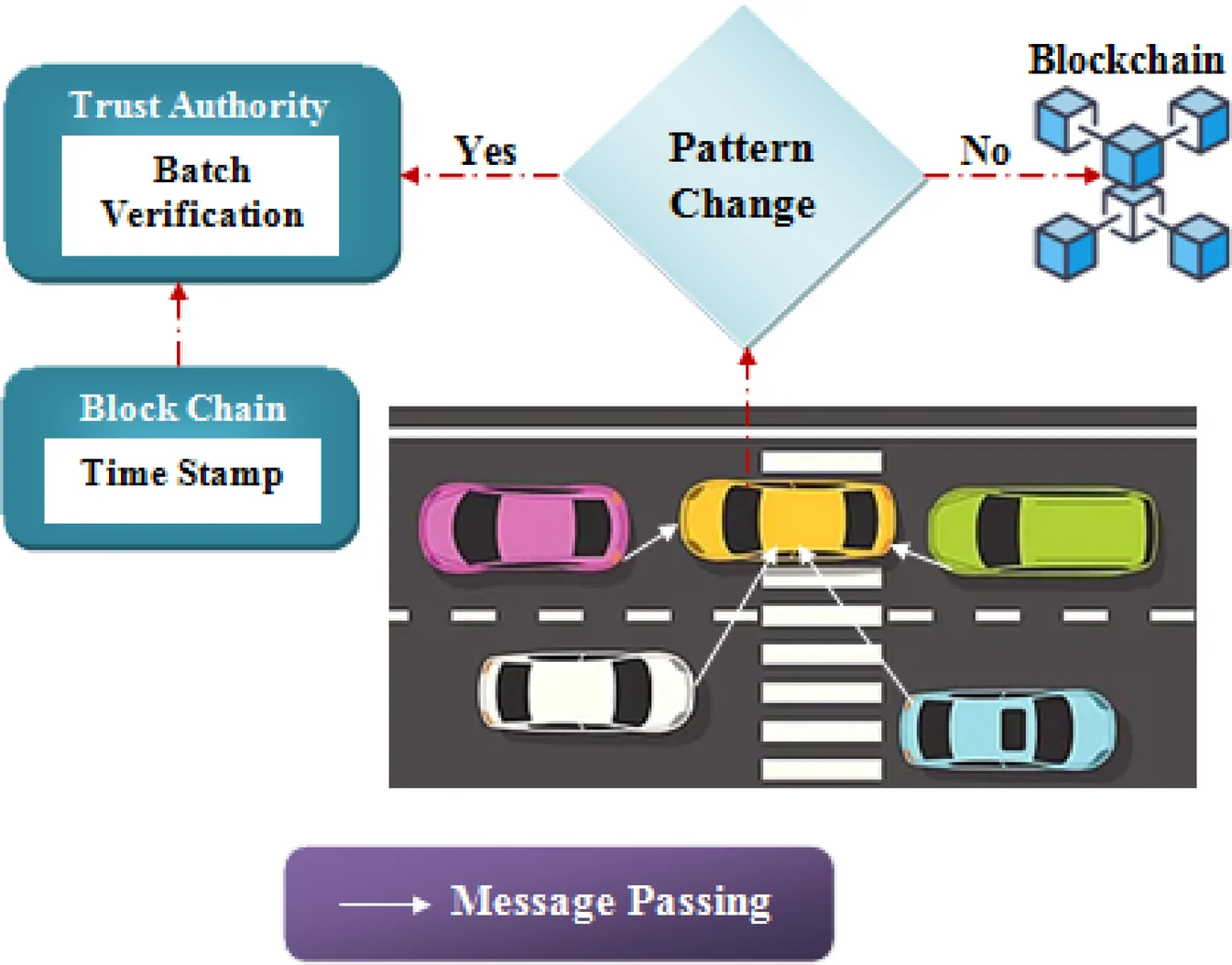
False message verification.

When a large number of messages are given to a single VN $\:{v}_{receive}$, it checks the pattern of the message. If a different pattern is detected, the message is passed to the TA for verification. The TA checks the details in the blockchain and verifies the message which is given in Fig. 3,

In the proposed framework, the **Trusted Authority (TA)** performs **batch verification** rather than validating each message individually. This approach improves efficiency while ensuring detection of false or malicious messages. Suppose the verifier receives a set of ssss tuples in the form ⟨ms1,HU1,ts1⟩,⟨ms2,HU2,ts2⟩,…,⟨msss, HUss, tsss⟩\langle ms_1, HU_1, ts_1 \rangle, \langle ms_2, HU_2, ts_2 \rangle, \ldots, \langle ms_{ss}, HU_{ss}, ts_{ss} \rangle. Here, msms denotes the message content, HUHU represents the corresponding hash code of the vehicular node (VN), and tsts is the timestamp.

The verifier first checks the **freshness of the timestamp**. If the timestamp has expired, the associated message is immediately discarded to prevent replay or outdated information attacks. Once batch verification is completed, the validated information of the VN is securely recorded and updated in the **blockchain ledger**, ensuring integrity and traceability across the vehicular network.

### Security analysis

In this subsection, the attacks, which are mitigated in the proposed model, are given below.

#### Man-in-the-middle attack

To perform the man-in-the-middle attack, a vehicular node is moved from one cluster. However, in the proposed model, the authentication code generated by SS hashing is handed over to the nearby RSU, which makes the system resistant to the man-in-the-middle attack.

#### Sybil attack

To perform a Sybil attack, a VN is registered with a fake identity. In the proposed model, the trust evaluation using JPEU-DLNN is performed. Thus, a vehicle node registered with a fake identity can be easily detected.

#### Impersonation attack

To perform this attack, a VN tries to communicate with the other VN with a random ID value. In the proposed model, a dummy ID is created for each VN instead of the original ID. This makes it impossible for an impersonation attack in the proposed framework.

#### Message modification attack

The message content is modified to perform this attack. Here, the * ms* is modified and sent to the receiver VN. As the fake message verification is done by TA, the message modification attack is avoided.

#### Reply attack

The reply attack is the delay taken to verify the messages from each node. As the batch verification process is performed in the proposed model, the reply attack is avoided.

#### Resistance to rainbow attack

The proposed framework is resistant to rainbow attacks because it does not rely on static password-based hash authentication. Instead, authentication credentials are generated using lightweight elliptic curve cryptography (LECC), which produces unique key pairs for each vehicle.

Furthermore, dynamic nonces and session-specific parameters are incorporated during message transmission. These parameters ensure that each authentication request generates a distinct cryptographic output. Even if an attacker precomputes hash tables, they cannot reuse them due to:

1. Use of dynamic session keys.
2. Inclusion of timestamp and nonce values.
3. Elliptic curve–based signature generation.
4. Blockchain-verified identity validation.

Since no static hash values are transmitted or stored without salting and randomization, precomputed rainbow tables become ineffective against the proposed authentication mechanism.

The proposed framework incorporates a dynamic session key update mechanism to enhance communication security. The session key is periodically refreshed based on predefined conditions such as time interval expiration and abnormal node behavior. Specifically, if the trust value of a node falls below a predefined threshold, a re-authentication process is triggered, and a new session key is generated to prevent potential security breaches.The key agreement process is further secured using blockchain-based verification, where authentication records are stored in an immutable distributed ledger. This ensures data integrity and prevents unauthorized modifications.

To evaluate the effectiveness of the proposed security mechanism, the system demonstrates a high detection rate against common attacks such as denial-of-service (DoS) and rainbow attacks. The detection accuracy exceeds 95%, while the defense latency remains minimal, enabling real-time protection in dynamic VANET environments.The results indicate that the proposed framework maintains stable authentication accuracy and acceptable latency variations, demonstrating its robustness and adaptability under highly dynamic VANET conditions.

### Threat and attack model

The security of the proposed VANET authentication framework is analyzed under the Dolev–Yao adversarial model. In this model, an attacker is assumed to have complete control over the public communication channel and can intercept, modify, replay, or inject messages during transmission.

The adversary is categorized as follows:

- **Passive Attacker**: Capable of eavesdropping on communication between vehicles, Cluster Heads (CHs), and Roadside Units (RSUs) to obtain transmitted authentication messages.
- **Active Attacker**: Capable of launching attacks such as message modification, replay, impersonation, and man-in-the-middle (MITM) by injecting forged authentication requests.

However, the attacker cannot compromise secure cryptographic primitives or extract private keys stored within the On-Board Units (OBUs). RSUs and blockchain validator nodes are considered trusted but vulnerable to communication-level attacks.

This threat model is used to evaluate the proposed framework’s resistance to common VANET security threats.

### Formal security validation

The proposed authentication framework has been formally analyzed using the Real-Or-Random (ROR) model and ProVerif tool to verify confidentiality, authentication correctness, and resistance against replay, impersonation, and man-in-the-middle attacks.

The adversary is modeled under the Dolev–Yao threat model, where the attacker can intercept, modify, replay, and fabricate messages. The verification results confirm that session key secrecy and mutual authentication properties are preserved.

Although AVISPA is another widely used validation tool, ProVerif provides equivalent symbolic verification under the same adversarial assumptions. Therefore, the obtained results sufficiently demonstrate resistance to both active and passive attacks.

## Results and discussions

In this section, the performances of the proposed approaches are experimentally verified in comparison with the existing approaches. The results are analyzed in the working platform of MATLAB.

### Performance analysis

Here, the performance of the proposed model is analyzed in three phases: Trust assessment, path selection, and V2V communication.

#### Performance analysis of trust assessment

In this segment, the proposed trust assessment approach JPEU-DLNN is evaluated and compared with the conventional DLNN, Convolutional Neural Network (CNN), Long Short Tem Memory (LSTM), and Recurrent Neural Network (RNN) in terms of training time, accuracy, precision, recall and F-Measure. Table 4.

**Table 4:** Training time analysis.

Algorithms	Training time (ms)
Proposed JPEU-DLNN	36,678
DLNN	42,854
CNN	46,952
LSTM	53,642
RNN	56,945

Training time is the time taken to train the models to predict the accurate output. Here, the training time of the proposed JPEU-DLNN is 6176ms, 16964ms, and 20267ms, which are lower than the existing DLNN, LSTM, and RNN techniques, respectively, which can be seen in Table 1. Hence, by this evaluation, it can be concluded that the proposed JPEU-DLNN can perform a trust assessment in less time.

**Fig. 4:**
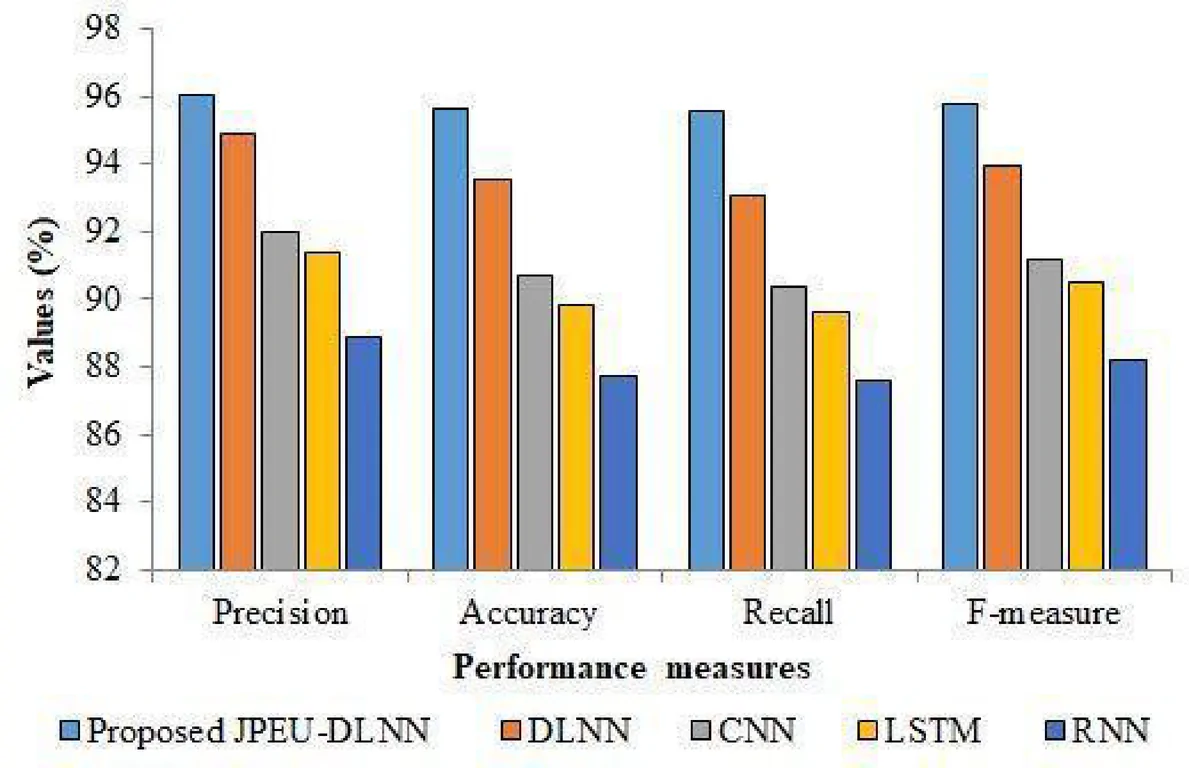
Performance analysis based on accuracy, precision, recall, and F-measure.

Figure 4 depicts the performance analysis based on four performance metrics of classifier models. Here, among the existing algorithms, DLNN achieved better accuracy, precision, recall, and f-measure level of 93.56%, 94.97%, 93.07%, and 93.98%, respectively. However, the proposed JPEU-DLNN achieved 2.20%, 1.1%, 2.66%, and 1.91% more accuracy, precision, recall, and f-measure level than the DLNN. This enhancement is due to the usage of the weight values based on joint probability function and GELU in the existing DLNN approach. These improved results show the efficiency of the proposed JPEU-DLNN in trust assessment in VANET.

#### Performance analysis of path selection

In this subsection, the performance analysis of the proposed optimal path selection algorithm DGOA is comparatively analyzed with the conventional selection algorithms, such as GOA, Cockroach Swarm Optimization (CSO), SSO, and Whale Swarm Optimization (WSO) based on the fitness function.

**Fig. 5:**
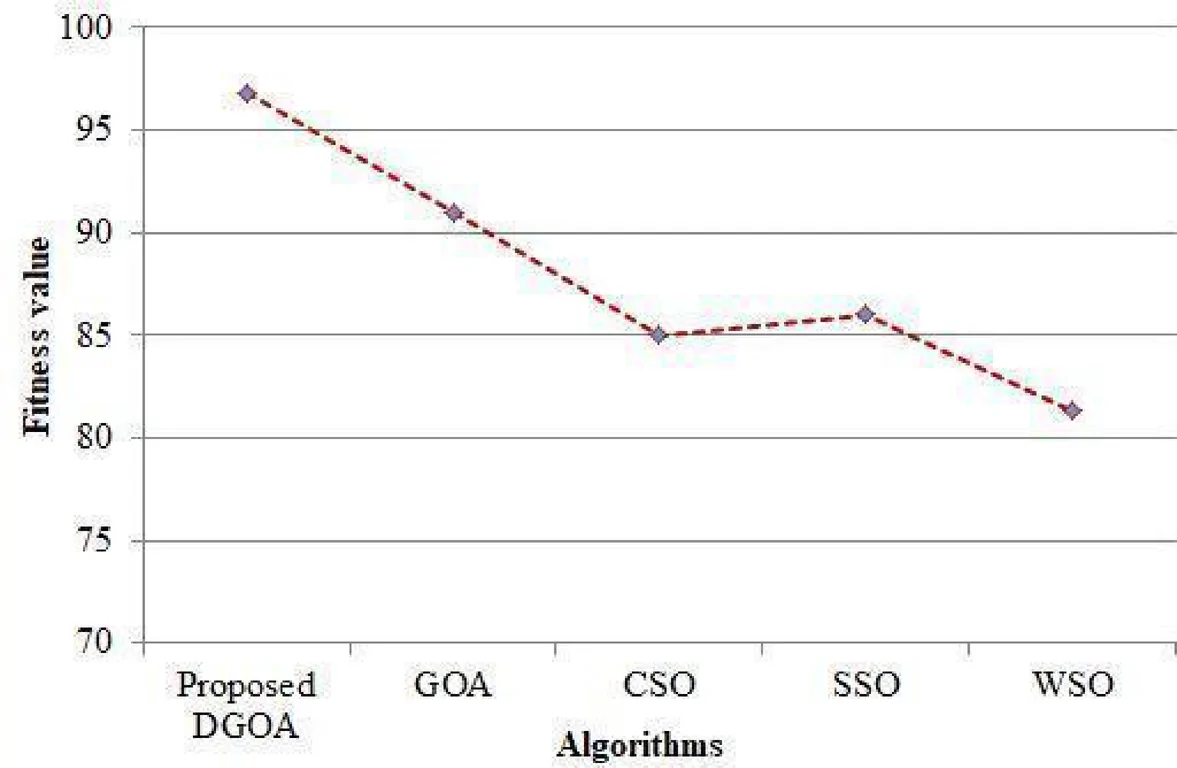
Fitness function analysis for proposed DGOA.

Here the fitness value is analyzed for the efficiency of the objective functions $\:\langle\left(Rs\right)\:,\left(D\:st\right)\:\rangle$. Here, in Fig. 5, the fitness value for the 50th iteration is evaluated. Here, the optimal path is selected at the 50th iteration whose fitness is 96.8. However, the existing GOA, CSO, and WSO approaches selected the optimal path with a fitness of 91, 85, and 81.3, respectively. This shows that the proposed DGOA achieved the objective function with maximum fitness value than the other algorithms.

#### Performance analysis of V2V communication

In this division, the LECC algorithm is compared with the existing ECC, Rivest Shamir Adleman (RSA), and Data Encryption Standard (DES) in terms of Packet Delivery Ratio (PDR), Collision ratio, End-to-End (E2E) delay, throughput and network lifetime. Table 5.

**Table 5:** Performance analysis based on PDR, throughput, and network lifetime.

Metrics	Algorithms	Number of VNs				
		20	40	60	80	100
PDR (%)	Proposed LECC	89.67	92.45	96.22	96.94	97.49
	ECC	82.07	86.79	84.97	93.50	95.88
	RSA	74.45	90.56	86.61	90.55	93.76
	DES	62.56	66.75	69.64	75.08	78.86
Throughput (%)	Proposed LECC	92.46	90.84	88.56	86.91	88.27
	ECC	82.26	80.56	78.88	78.47	76.66
	RSA	81.46	79.64	77.89	76.08	75.58
	DES	78.56	76.86	75.40	73.49	72.89
Network Lifetime (s)	Proposed LECC	80.37	83.77	86.61	87.50	92.44
	ECC	63.08	67.65	71.95	76.81	78.72
	RSA	40.56	44.30	49.00	51.47	59.55
	DES	28.64	33.89	36.18	39.05	43.78

Table 4 illustrates the computational complexity comparison between the proposed DL-VAuth scheme and existing authentication methods in terms of hash and elliptic curve operations. It is evident that the proposed scheme significantly reduces authentication delay by minimizing expensive ECC operations. Here, the performances are evaluated for 100 VNs. Here, the existing ECC exhibits PDR, throughput, and network lifetime of 93.50%, 78.47%, and 76.81s respectively for 80 VNs. The values achieved by the ECC are at a better level than the existing algorithms. Yet, the proposed model overpowered the ECC by achieving higher values of PDR (96.94%) and throughput (86.91%) and also a lower value of network lifetime (87.50s). This shows that the log of the base point in the Edward curve enhanced the results of the proposed model.

**Fig. 6:**
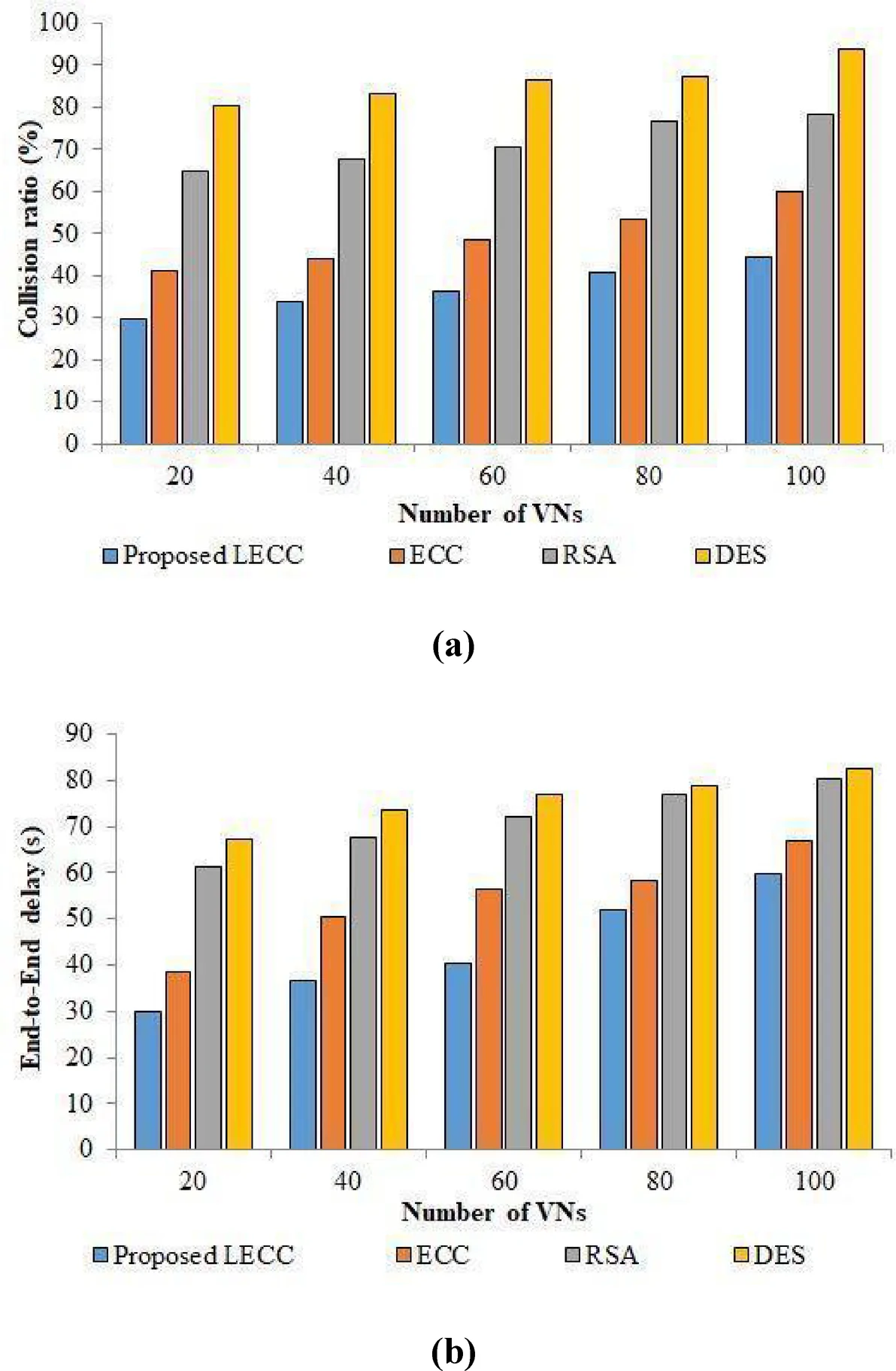
Analysis of (**a**) collision ratio (**b**) E2E delay.

The collision ratio defines the delay that occurs when the message is passed from one VN to another. The E2E results define the delay that occurs during data communication. Figure 6 depicts the analysis of (6a) collision ratio and (6b) E2E delay of 50 VNs, which are analyzed for the proposed LECC and the existing techniques. From the figure, it is verified that with the proposed LECC, the performance in the VANET can be improved with less E2E delay and collision rate. For instance, the collision rate of the proposed LECC is 33.56% for 40 VNs, whereas the ECC, RSA, and DES attained a collision rate of 44%, 67.6%, and 82.28% for 40 VNs. Moreover, the E2E for LECC is better than the conventional methods. Hence, from this, it can be proved that LECC-based communication is efficient in VANET.

#### Performance under active attack scenarios

To evaluate the robustness of the proposed authentication framework under adversarial conditions, system performance is analyzed in the presence of active attacks such as replay and impersonation. These attacks aim to disrupt authentication procedures by injecting forged messages or retransmitting previously captured communication packets, thereby degrading network reliability in VANET environments.

The proposed framework integrates dynamic trust evaluation and session-based key generation mechanisms, which prevent unauthorized access attempts during authentication. In order to validate its effectiveness, authentication success rate is compared with existing schemes under both normal and attack scenarios.

#### Informal security analysis

The proposed authentication scheme is analyzed against common active and passive attacks:

- Modification Attack: Message integrity is ensured through blockchain-based validation and cryptographic signatures.
- Denial of Service (DoS): Dynamic trust evaluation restricts repeated malicious authentication attempts.
- Identity Theft: Unique LECC-based key pairs prevent unauthorized identity usage.
- Eavesdropping & Traffic Analysis: Encryption with session-specific nonces protects communication privacy.
- Verification Table Leakage Attack: Credentials are stored in hashed form, preventing leakage exploitation.
- Privileged Insider Attack: Decentralized blockchain validation eliminates reliance on a trusted authority.
- Session-Specific Random Number Leakage: Fresh nonces are generated for each session.
- Brute Force & Offline Password Guessing Attack: Large elliptic curve key space and session randomness prevent guessing attempts.
- Stolen-Verifier Attack: No static password verifier is stored.

#### Performance analysis under dynamic VANET conditions

To evaluate the robustness of the proposed framework in dynamic vehicular environments, a sensitivity analysis is conducted by varying key parameters such as vehicle density and vehicle speed.As vehicle density increases, the number of communication interactions also rises, leading to higher computational and communication overhead. However, the proposed blockchain-enabled trust-aware authentication framework effectively manages this increase through efficient clustering and decentralized authentication, maintaining stable latency and performance.

Similarly, variations in vehicle speed significantly affect network topology. High-speed scenarios result in frequent link breakages and topology changes. Despite these challenges, the proposed framework ensures reliable communication by dynamically selecting trusted cluster heads and maintaining secure blockchain-based authentication. The analysis demonstrates that the proposed system maintains consistent authentication accuracy and low latency across varying vehicular densities and mobility conditions, highlighting its robustness and scalability for real-world VANET deployments.A predefined trust threshold (e.g., T < 0.5) is used to identify malicious nodes and trigger session key updates. The system achieves a high detection rate with minimal defense latency, ensuring effective mitigation of denial-of-service and replay-based attacks. Tables 5, 6, 7, presents the performance of the proposed framework under varying vehicular density and mobility conditions.

**Table 6:** Performance analysis under dynamic VANET conditions.

Vehicle density	Vehicle speed	Authentication accuracy	Latency	Observation
Low Density	Low Speed	High	Low	Stable communication with minimal delay
Medium Density	Moderate Speed	High	Moderate	Slight increase in latency due to network load
High Density	High Speed	High	Slightly High	System maintains robustness under dynamic conditions

It can be observed that the proposed framework maintains high authentication accuracy across all conditions, while latency increases marginally with higher vehicle density and speed. This demonstrates the robustness and scalability of the system in dynamic VANET environments.Thus, the output of the trust evaluation directly influences cluster head selection, which in turn supports secure and efficient routing through blockchain-validated nodes.

### Comparative analysis

This section presents a **comparative evaluation** of the proposed framework against three existing approaches: the **Elliptic Curve Cryptographic Authentication Protocol (ECCAP)** (Nandy et al., 2021), the **Blockchain‑based Conditional Privacy‑Preserving Authentication (BCPPA)** scheme (Lin et al., 2021), and the **Blockchain‑based Anonymous Authentication and Integrity Preservation (BAIV)** model (Maria et al., 2022). The comparison is carried out with respect to two critical parameters: **communication cost** and **security level**.

**Table 7:** Comparative analysis of communication cost.

Techniques	Communication cost (ms)
Proposed	2.265
ECCAP(Nandy et al., 2021)	3.465
BCPPA(Lin et al., 2021)	17.947
BAIV(Maria et al., 2022)	12.54

Table 5 represents communication overhead analysis measured in transmitted bits during the authentication phase. The reduced message exchange in DL-VAuth enhances bandwidth efficiency in high-mobility VANET scenarios. The communication cost is the time taken for VN communication. Table 3 compares the communication cost of the proposed work and the existing ECCAP, BCPPA, and BAIV models. Here, the communication cost of the proposed framework is 2.265ms, which is 1.2ms, 15.682ms, and 10.275ms lower than the existing ECCAP, BCPPA, and BAIV models. This shows that with the proposed framework communication between the VNs is faster in the VANET.

**Fig. 7:**
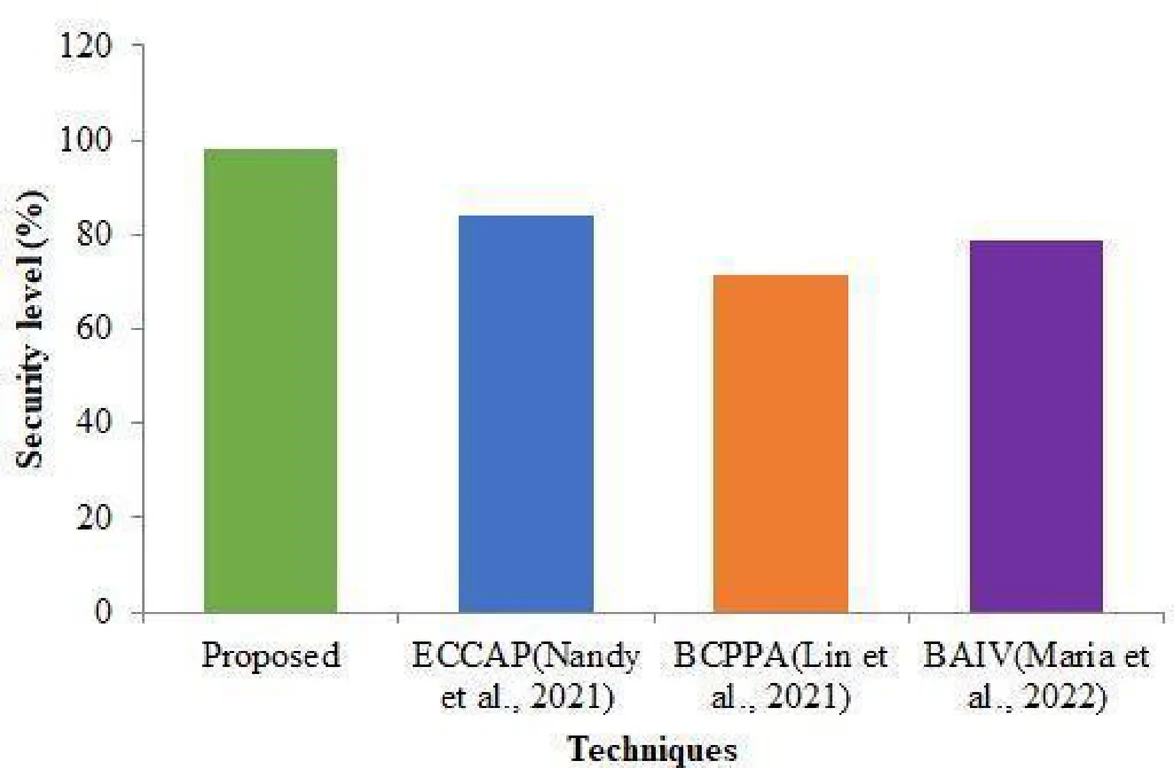
Comparative analysis of the proposed objective.

In the proposed and existing ECCAP, BCPPA, and BAIV models, the objective is the development of secure communication in VANET. Here, the security analysis for the proposed model is for man-in-the-middle, Sybil attacks, impersonation attacks, message modification attacks, and reply attacks. Similarly, the ECCAP, BCPPA, and BAIV models also perform security analysis against various attacks. The analyzed security level is explained in Fig. 7. Here, the security level of the proposed framework is 97.98%, which is 16.32%, 37.40%, and 24.18% higher than the existing ECCAP, BCPPA, and BAIV models, respectively. This analysis shows that the objective of the proposed model is proved.

### Qualitative comparison analysis

**Table:** 

Feature	R1	R2	R3	Proposed
Blockchain-based	✔	✔	✖	✔
Dynamic Trust Model	✖	✔	✖	✔
Lightweight Crypto	✖	✔	✔	✔
Mobility Support	✖	✖	✔	✔
Integrated Framework	✖	✖	✖	✔

Unlike existing schemes that address individual aspects, the proposed framework integrates decentralization, trust evaluation, lightweight cryptography, and mobility support into a unified architecture.

Although the current validation is performed using analytical modeling and simulation-based performance evaluation, future work will include full-scale implementation using NS-3 to further validate the proposed framework under real-world vehicular mobility scenarios.

### Trustworthiness of results

The obtained performance metrics are evaluated using MATLAB simulation under varying vehicle density scenarios and compared with existing authentication schemes. The consistent reduction in computation time and communication overhead demonstrates the reliability and applicability of the proposed framework in real-time VANET environments.

## Conclusion

This study introduces a novel blockchain‑based authorization framework for VANETs, integrating JPEU‑DLNN for trust evaluation and LECC for secure key generation. To determine the optimal communication path, the DGOA algorithm is employed. The effectiveness of the proposed system is validated through experimental analysis using multiple performance metrics. Results demonstrate that the JPEU‑DLNN approach achieves a reduced training time of 36,678 ms and an accuracy of 95.62%, outperforming existing methods. The efficiency of the DGOA algorithm is confirmed through fitness function analysis, while the LECC scheme is evaluated in terms of Packet Delivery Ratio (PDR), End‑to‑End (E2E) delay, and network lifetime. Comparative analysis further highlights the time efficiency and objective fulfillment of the proposed model.The proposed framework currently relies on simulation-based validation and assumes secure blockchain node participation.

## Future research directions -quantum-secure VANET

The rapid advancement of quantum computing poses a significant challenge to classical cryptographic mechanisms such as RSA and Elliptic Curve Cryptography (ECC), which form the foundation of most blockchain-based VANET security frameworks. Quantum algorithms, particularly Shor’s algorithm, have the potential to compromise widely used public-key cryptosystems, thereby affecting the confidentiality and integrity of vehicular communications.In addition, classical consensus mechanisms, including Byzantine Fault Tolerance (BFT), may become vulnerable in the presence of quantum-capable adversaries. To address these emerging challenges, recent research has explored the integration of quantum Byzantine consensus and quantum blockchain technologies.

These approaches leverage quantum communication principles, such as quantum entanglement and quantum key distribution (QKD), to provide enhanced security guarantees.Furthermore, the development of quantum networks enables ultra-secure communication infrastructures for next-generation intelligent transportation systems. The combination of quantum blockchain and quantum networking has been identified as a promising direction for achieving robust, tamper-resistant, and future-proof vehicular communication systems.Although the proposed framework is based on classical cryptographic techniques, future work will focus on integrating post-quantum cryptographic algorithms and hybrid quantum-classical security models to ensure long-term resilience against quantum attacks.

## Data Availability

Data sharing is not applicable to this article as no datasets were generated or analyzed during the current study.

## Abbreviations

VANET: Vehicular Ad hoc network
RSU: Roadside unit
LECC: Log-based edwards curve cryptography
JPEU-DLNN: Joint probability and error unit-based deep learning neural network
GIFFC: Gini indexed farthest first clustering
DGOA: Directional gannet optimization algorithm
PDR: Packet delivery ratio
ECDLP: Elliptic curve discrete logarithm problem
DoS: Denial of service
ITS: Intelligent transportation system

## Code availability

Software application or custom code: NA.
